# MicroRNA molecular profiling identifies potential signaling pathways conferring resistance to chemoradiation in locally-advanced rectal adenocarcinoma

**DOI:** 10.18632/oncotarget.25652

**Published:** 2018-06-22

**Authors:** Cory Pettit, Amy Webb, Steve Walston, Moumita Chatterjee, Wei Chen, Wendy Frankel, Carlo Croce, Terence M. Williams

**Affiliations:** ^1^ The Ohio State University Medical Center, Arthur G. James Comprehensive Cancer Center and Richard J. Solove Research Institute, Columbus, OH 43210, USA

**Keywords:** miRNA, rectal cancer, chemotherapy, radiation, biomarker

## Abstract

**Purpose:**

There has been growing interest in using chemoradiation (CRT) for non-operative management of rectal cancer, and identifying patients who might benefit most from this approach is crucial. This study identified miRNAs (miRs) associated with clinical outcomes and treatment resistance by evaluating both pre- and post-CRT expression profiles.

**Methods:**

Forty patients, 9 with pathologic complete response (pCR) and 31 with pathologic incomplete response (pIR) were included. MicroRNA was extracted from 40 pre-therapy tumor samples and 31 post-chemoradiation surgical samples with pathologic incomplete response (pIR). A generalized linear model was used to identify miRs associated with pCR. A linear mixed effects model was used to identify miRs differentially expressed before and after treatment. miR expression was dichotomized at the mean and clinical outcomes were evaluated using Cox proportional hazard modeling.

**Results:**

Nine miRs were associated with pCR (p<0.05), but none were significant after false discovery rate correction. Among patients with pIR, 68 miRs were differentially expressed between the pre and post-CRT groups (FDR p<0.05). Ingenuity pathway analysis (IPA) demonstrated multiple signaling networks associated with pIR, including p38MAPK, TP53, AKT, IL-6, and RAS. Increased let-7b was correlated with increased distant metastasis (DM), worse relapse-free survival (RFS), and worse overall survival (OS) (p<0.05).

**Conclusions:**

No miRs were significantly correlated with pCR. We identified miRs that were differentially expressed between pre- and post-CRT tumor samples, and these miRs implicated multiple signaling pathways that may confer resistance to CRT. In addition, we identified an association between increased let-7b and worse clinical outcomes (DM, DFS, OS).

## INTRODUCTION

Rectal adenocarcinoma accounts for almost 1/3 of all colorectal cancer, with approximately 40,000 new cases diagnosed each year, and rectal cancer is a major cause of cancer morbidity and mortality in the United States [1]. Current treatment regimens include neo-adjuvant (pre-operative) chemoradiation followed by surgical removal for locally advanced rectal cancer (T3-4 or node positive disease) [2, 3]. Typically, curative surgical resection occurs between 6-12 weeks after chemoradiation [4]. In 25-50% of cases, a complete clinical response (cCR) is observed after chemoradiation prior to surgery (at endoscopic assessment), or at the time of surgery [5]. Upon pathologic examination of the surgical specimen, however, only 10-20% of cases demonstrate a pathological complete response (pCR) [6, 7]. Given that the number of pelvic recurrences after pCR are very low, there has been growing interest in finding biomarkers that may predict which patients will have a complete clinical or pathologic response to chemoradiation, or who are at decreased risk of pelvic recurrence after chemoradiation [8, 9]. In addition, there has been a growing movement to consider offering patients close surveillance after chemoradiation, termed non-operative management (NOM), by performing regular endoscopy and/or pelvic MRI scans to assess for recurrence [10]. While NOM is controversial, the discovery of biomarkers that reliably predict response and recurrence rates after chemoradiation offers the potential to better select patients for this strategy. This NOM approach could potentially spare patients invasive, life-changing surgeries (especially for patients requiring permanent colostomies), and improve their quality of life if the risk of pelvic recurrence is low. In those instances, surgery could be used as a salvage option for patients who do have a pelvic recurrence.

A class of biomarkers of growing interest is micro-RNA (miR) [11]. miRNA begins as pri-miRNA and resides in the nucleus. It is then cut into pre-miRNA and leaves the nucleus, after which it is processed into mature miRNA by the enzyme complex DICER. Mature miRNA is subsequently loaded onto the RISC complex, which delivers it to the mRNA transcripts that the particular miR sequence silences [12]. Certain miRs may exhibit an oncogenic or tumor suppressive effect depending on which genes or groups of genes they silence. Unlike mRNAs, miRs have a long half-life in the serum, and are not degraded. Therefore, miRs that are found to correlate with a specific response to chemoradiation may potentially be used as biomarkers to predict response, and guide therapeutic decision-making. Many studies on miRs and response to chemoradiation in locally-advanced rectal carcinoma (LARC) have been carried out, but few consistent gene expression patterns have been identified.

Previous studies have examined the relationship between expression of key miRs and response to chemoradiation [13, 14]. However, most of these studies only looked at pCR, and not clinical response to therapy. To date, a few studies have examined clinical response, but not long term clinical outcomes such as relapse-free survival and overall survival. A 2014 study by Lopes-Ramos assessed clinical response via rectal exam, pelvic MRI, proctoscopy, and CEA levels [15]. In addition, a 2013 study by Hotchi et al. used RECIST to examine clinical response to therapy in addition to pCR [16]. Most of the previous studies have examined the independent ability of certain miRs to predict response to therapy, but a few have found predictive groups or “signatures” of miR expression that correlate with therapeutic response. A study by Kheirelseid et al. found a signature of three miRs that correlated with increased pCR [17], and a study by Scarpati et al. found a separate signature that correlated with response [18]. Finally, only one study today has examined the expression levels of miRs before and after therapy. In this study, Svoboda et al. found two miRs to be up-regulated after chemoradiation therapy [19].

In this study, we have performed miRNA profiling on 40 patients with locally advanced rectal cancer who all received chemoradiation prior to surgery. We attempted to identify pre-treatment miRNAs associated with pCR and clinical outcomes (relapse-free survival, overall survival), and identified let-7b as associated with these clinical outcomes. In addition, we performed an analysis of miRNAs that were changed before and after chemoradiation, in order to identify pathways that might be conferring resistance to therapy and tumor survival. We found significant changes in miRNA expression patterns after chemoradiation, and pathway analysis implicates a number of pro-proliferative and survival pathways, including AKT, TP53, p38MAPK, IL-6, and RAS.

## RESULTS

**Figure 1:**
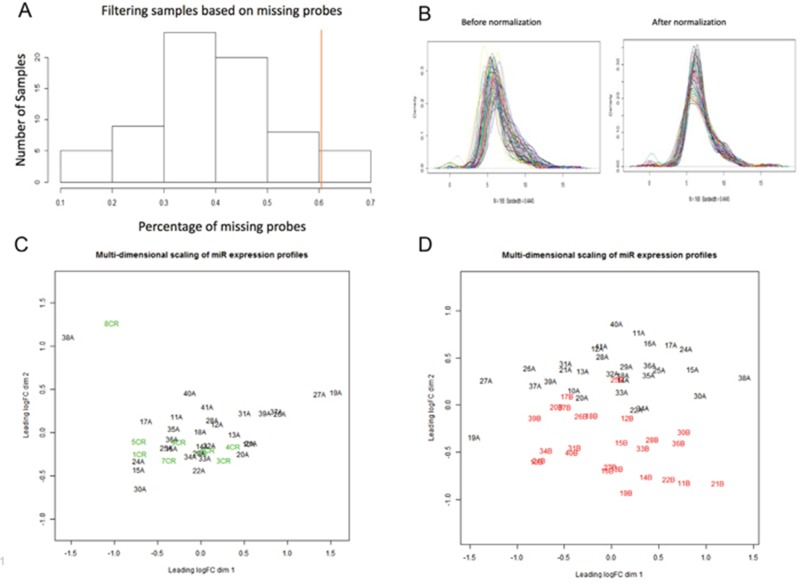
Multi-dimensional scaling of pre-therapy and post-therapy samples for miRNA molecular profiling **(A)** Histogram displaying filtering of samples based on missing probes. We removed 5 patient samples (to the right of the red line) based on >60% missing probes. **(B)** Density distribution of miRNAs (n=168) for each patient sample before (left) and after (right) normalization. Normalization standardizes the mean and smooths distribution of miRNAs (bandwidth= 0.445). **(C)** Multi-dimensional scaling (MDS) of pre-therapy miR profiles in patients with pCR (green or “CR”) and pre-therapy miR profiles in patients with pIR (black, “A samples”). As observed, there is no significant separation in pre-therapy profiles between pCR and pIR patients. Distances on the plot demonstrate the log2-fold changes between the samples. **(D)** Multi-dimensional scaling (MDS) of pre-therapy miR profiles in patients pIR (black, “A samples”) and post-therapy miR profiles in patients with pIR (red or “B samples”). A significant separation is observed between pre-therapy and post-therapy miR profiles in pIR patients. and post-therapy miR profiles in patients with pIR.

Forty patients had sufficient pre-treatment (diagnostic biopsy) tissue for this study. Nine patients had pathologic complete response (pCR), and therefore had no post-therapy tumor to analyze. Thirty-one patients had pathologic incomplete response (pIR) and had both pre-treatment and surgical tissue available for analysis. NanoString miRNAome profiling on ~800 miRs was performed. After the results were normalized and filtered, the minimum number of counts for a miR was 32 counts, and the maximum was 66,418 counts. The average number of counts per miR was 700. Five patient samples were excluded after statistical filtering (Figure 1A), based on >60% of miRNA probes missing. These 5 samples all occurred in the surgical samples from the pIR group, leaving suitable miR expression data for 26 patients. Then we performed normalization and filtering of the miRNAs on the patient samples, resulting in a total of 168 miRNAs that were analyzable (Figure 1B). In order to determine associations between pre-treatment samples in both the pCR and pIR groups, as well as the pIR pre-treatment and post-treatment samples, we performed multidimensional scaling (MDS). In MDS analyses, samples are plotted on a two-dimensional scatterplot so that distances on the plot approximate the typical (root-mean-square) log2-fold changes between the samples. As shown in Figure 1C, there did not appear to be significant separation between the pCR and pIR groups based on expression of pre-therapy miRNAs. However, we noted significant separation between pre- and post-therapy tissues in the pIR group (Figure 1D).

First, pre-therapy samples of pCR patients were compared to pre-therapy samples of non-responders (pIR), to examine if there was a differential expression pattern of miRs between them and allow the ability to generate a miR signature that could predict clinical/pathologic complete response to chemoradiation. Initially, 9 miRs were found to be differentially expressed between the two groups, with a p value <0.05. However, after correcting for false discovery rate (FDR), none of these miRs had an FDR p< 0.05, and therefore were not truly statistically significant.

**Figure 2:**
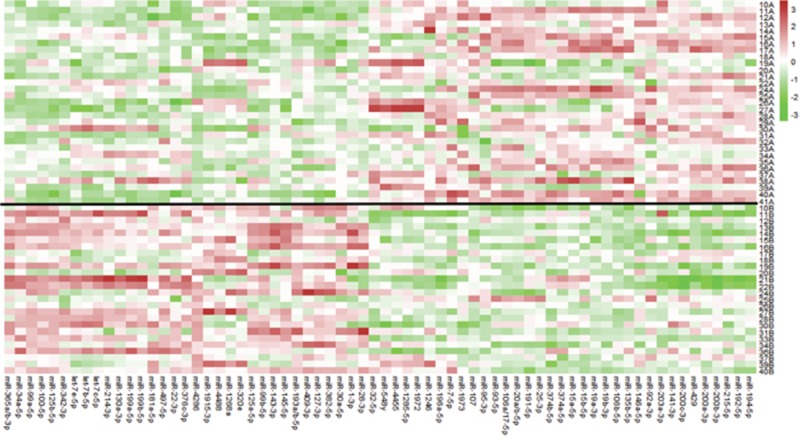
Heat map of differentially expressed miRNAs between pre-therapy and post-therapy tumor samples in patients with pIR 68 miRNAs were identified (bottom axis) using a linear mixed effect model analysis (using FDR<0.05). The red colors indicate higher expression for a particular miRNA, while the green colors represent lower expression. The black line divides the patient samples between pre-therapy (above black line, “A” samples) and post-therapy (below black line, “B” samples) samples. For example, miR-4286 is predominantly green in pre-therapy samples and red in post-therapy samples, indicating a significant increase in expression in post-therapy samples. miRNA expression values are normalized to the average value (0) using z-score normalization methods, where over-expression of a particular miR is shown in red, no change shown in white, and under-expression shown as green.

**Figure 3:**
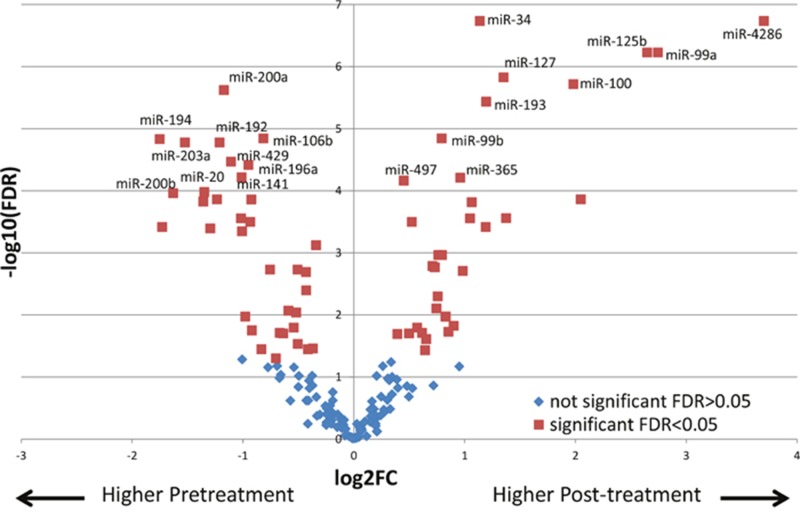
Volcano plot displaying differentially expressed miRNAs between pre-therapy to post-therapy tissues miRNAs higher in post-treatment samples (right side) and miRNAs lower in post-treatment samples (left side) are shown. miRNAs that were significantly differentially expressed after FDR correction (p<0.05) are shown as red squares. In addition, miRNAs that had a FDR p-value<10^−4^ are labeled. X axis depicts log_2_ expression fold change, while Y axis depicts log_10_ FDR p-value.

Next, we sought to determine the changes in miRNA expression after chemoradiation in the incomplete responders, which could potentially provide insight into molecular pathways conferring resistance to therapy. Pre-therapy samples of pIR patients were compared to the post-therapy samples of these same patients (n=26). Using a linear mixed effect model where the pre-therapy and post-therapy samples from the same patient are matched and included in the model as a random effect, 68 miRs were found to be differentially expressed between the two groups with an FDR p<0.05 (Supplementary Table 1). A heat map demonstrating the differential expression between both groups with the 68 miRs is shown in Figure 2. For example, for miR-4286, there was significantly higher expression in post-therapy tissues as compared to pre-therapy tissues (~13-fold). In order to compare the directionality of expression and significance level of miRNAs in pre-treatment vs post-treatment tumor tissue, we plotted individual miRNAs as a function of their log_2_ expression fold change with their log_10_ FDR p-value using a volcano plot, as depicted in Figure 3. miRNAs that were significantly differentially expressed after FDR correction (p<0.05) are shown as red squares. In addition, miRNAs that had a FDR p-value<10^−4^ are labeled. For example, we found dramatic and highly statistically-significant increases in miR-4286 in post-therapy tissue, as well as miR-125b, -99a, -100, -34, -127, and -193. Conversely, we identified significant down-regulation of miR-194, -200a, -203, -200b, -20, -192, -429, -196a, -141, and -106b in post-therapy tissue.

### Ingenuity pathway analysis

**Figure 4:**
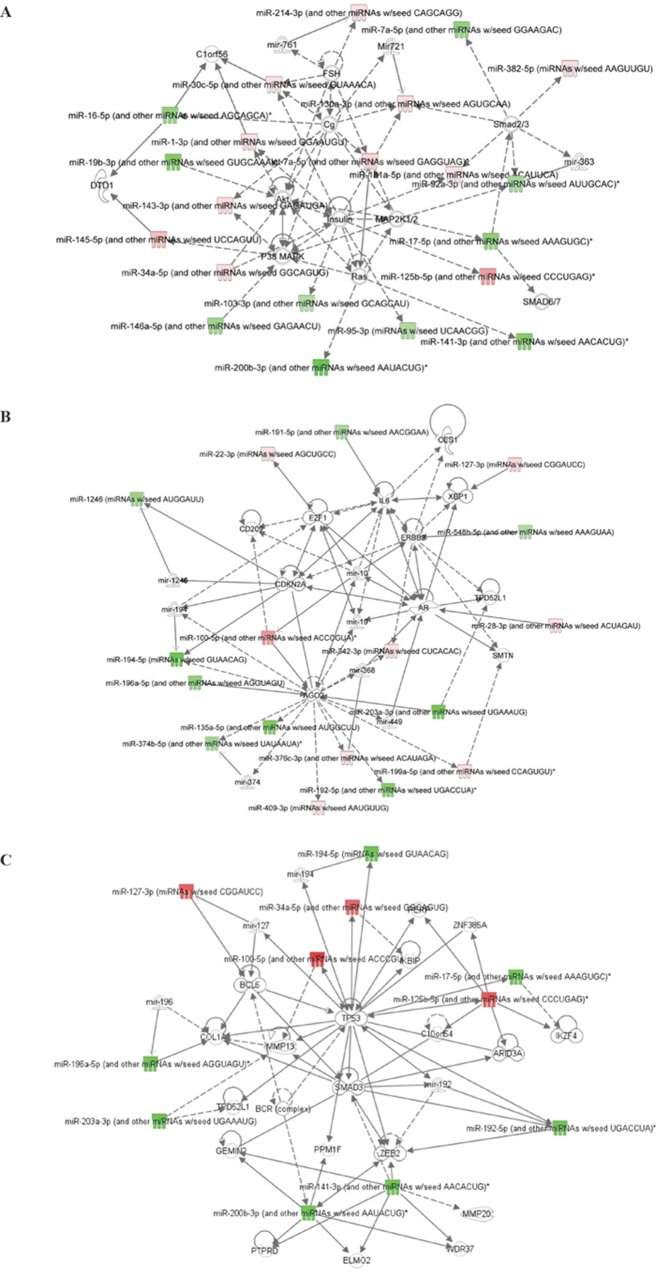
Ingenuity Pathway Analysis (IPA) of miRNAs differentially expressed between pre-therapy and post-therapy tumor samples from patients with pIR **(A)** Network #1 demonstrates potential associations between these miRNAs and p38 MAPK, Ras, MAP2K1/2, and Smad family proteins. **(B)** Network #2 demonstrates potential associations between these miRNAs and IL-6, ERBB2, E2F1, and CDKN2A. **(C)** Network #3 is a more stringent analysis of only miRs with an FDR <10^−4^, and included molecules such as TP53, ZEB2, Smad3, and Bcl6.

An Ingenuity Pathway Analysis (IPA) was carried out to try to identify networks that involve these differentially expressed miRs between pre-therapy and post-therapy tumor tissues in patients with pIR, and their related signaling pathways. The most significant network, with a score of 51, involved 21 miRs or related miR molecules, which were significantly associated in the literature with cancer and organismal injury (Table 1). This pathway involves several crucial signaling molecules linked with survival, proliferation, and therapy resistance, including Akt, Ras, p38 MAPK, MAP2K1/2, and the Smad family proteins (Figure 4A). A second significant network (score of 39) was identified that contained molecules such as IL-6, ERBB2, E2F1, and CDKN2A (Table 1, Figure 4B). Finally, for a more focused understanding of molecular pathways altered by chemoradiation, we performed a more stringent analysis of those labeled miRs in the volcano plot in Figure 3 that had an FDR <10^−4^. This 3rd network had a score of 31, and was very similar to network 2, as it had 11 of the same miRs as network 2, but when compared to network 1, only 5 miRs were shared. In this network, TP53, Smad3, Bcl6, and Zeb2 signaling pathways were identified as potentially differentially activated based on miR expression (Figure 4C).

**Table 1:** Ingenuity Pathway Analysis of miRNAs differentially expressed between pre-therapy and post-therapy tumor tissues of patients with pIR

miR	Associated molecules in IPA	Other gene targets (miRTarBase)
***Network 1***		
miR-214	FSH	PTEN, MAP2K8, TP53, BCL2
miR-16	Cg, DTD1	BMI1, HMGA1
miR-30c	FSH, Cg	SMAD1, TGIF2
miR-7a	Smad 2/3, Ras	None discovered
miR-181	Smad 2/3, Insulin	BCL2, NOTCH2, NOTCH4, KRAS
miR-92	Smad 2/3, MAP2K	CDKN1C, TGFBR2, BCL2L11
miR-17	Smad 2/3, p38 MAPK	PTEN, E2F1, BCL2
miR-125b	Insulin	TP53, BMPR1B
miR-141	Ras	ZEB2, TGFB2, PTEN
miR-95	Insulin	SNX1, CELF2
miR-103	Insulin	CDK2, PTEN
miR-146a	P38 MAPK	CXCR4, BRCA1, BRCA2, FAF1
miR-200b	Ras	ZEB2,
miR-34a	Akt, Insulin	MYC, CDK4
miR-145	P38 MAPK	EGFR, HOXA9, STAT1
miR-143	P38 MAPK	KRAS, MYO6
miR-19b	Akt	PTEN
miR-130a	Smad 2/3, Cg, Insulin	SMAD4
miR-382	Smad2/3	PTEN
***Network 2***		
miR-191	IL-6	IL-1A, CDK6
miR-127	XBP1	BCL6
miR-548	ERBB2	ERBB2
miR-28	AR	CDKN1A, MAPK1, E2F6
miR-203a	TPD52L1	ABL1, BCL2L2, SMAD4, E2F1
miR-342	AGO2, ERBB2	BMP7, GEMIN4
miR-199	AGO2, SMTN	CD44, MAPK1
miR-192	AGO2	Zeb2
miR-409	AGO2	IFNG, RDX
miR-376	AGO2	CDK2, IGF1R
miR-135	AGO2	JAK2, APC, SMAD5
miR-374	AGO2	ATM
miR-196a	AGO2	CDKN1B, HOXA5, HOXA7
miR-194	AGO2, CDKN2A	SOX5, IGF1R
miR-100	AGO2, CD209	PLK1, FGFR3, ATM
miR-1246	CDKNA2	DYRK1A
miR-22	E2F1	STAT5A, CDKN1B
miR-135	AGO2	APC, JAK2, SMAD5, MYC

### Ingenuity pathway analysis- downstream effects predictor

An analysis within IPA termed “downstream effects predictor” was carried out to look at downstream functional effects of the 68 miRs that were significantly differentially expressed between pre-CRT and post-CRT samples. The only significant downstream effect resulting from this analysis that met the significant z score cutoff (<-2 or >2) was a predicted decrease in cell proliferation in the residual tumor cells after CRT (z-score −2.04). Supplementary Table 2 shows the list of miRs that contributed to this prediction. For example, let-7c, miR-145, miR-143, and miR-99a were noted to be increased after chemoradiation (positive fold change), and published findings support that increases in these miRs are associated with reductions in cell proliferation [20–23]. Conversely, miR-19b and miR-93 were noted to be decreased after chemoradiation (negative fold change), and published findings support that decreases in these miRs are associated with reductions in cell proliferation [24]. Thus, integrating the directionality of the effects for these 6 miRs supports a reduction in tumor cell proliferation in the post-treatment residual tumor samples. Also noted as downstream effects were “decreased metastasis of tumor cells”, “decreased tumor growth”, and “decreased angiogenesis”, but the scores ranged from −1.52 to −1.71 (borderline significant). In addition, the downstream effects predictor noted higher miR signatures consistent with “apoptosis of carcinoma cell lines” (z score range >1.49-1.90), but this result was also borderline significant.

### Correlation of miRs with clinical outcomes

**Figure 5:**
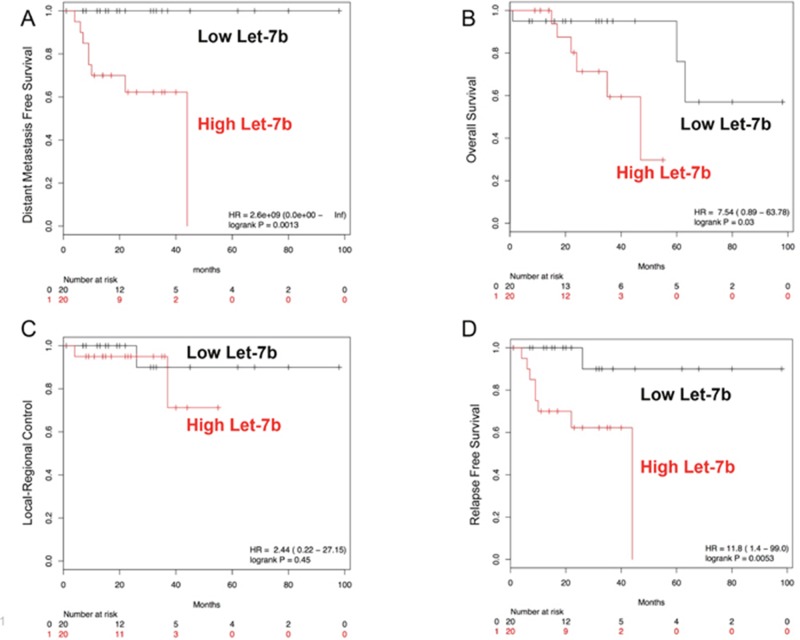
Kaplan-Maier survival plots for let-7b Higher pre-therapy levels of let-7b are associated with worse DMFS **(A)**, RFS **(C)**, and OS **(D)**. No statistically significant relationship between pre-therapy let-7b levels and LRC was found **(B)**.

We examined the role of some typical clinical variables on the clinical outcomes of these 40 patients. First, we examined gender, but found no associations between gender and overall survival (OS), relapse-free survival (RFS), or distant metastasis-free survival (DMFS). For pathologic tumor (pT) stage, we found that higher pT stage (3-4 vs 0-2) was significantly correlated with reduced overall survival (hazard ratio 9.6, 95% CI (1.2-78.8), log-rank p=0.01). For pathologic nodal (pN) stage, we found that node positive disease, was significantly associated with worse DMFS (hazard ratio 6.9, 95% CI (0.84-57.14), log-rank p=0.04). Next, we examined whether pre-treatment miRs could predict for clinical outcome. Candidate miRs were identified by Cox proportional hazard testing on clinical outcomes and validated by Kaplan-Maier survival curves. We identified let-7b as significantly associated with RFS and DMFS (Cox proportional hazards modeling p<0.05). We dichotomized miR expression about the geometric mean for each miR and generated survival curves for the various clinical events. We found that higher expression of let-7b was significantly associated with worse DMFS, RFS, and OS, but no significant difference in LRC (Figure 5A–5D). A multivariate analysis controlling for tumor stage, nodal stage, and age was then carried out. Including these clinical variables into the model, let-7b levels remained independently correlated with DMFS and RFS (p =0.029 and p=0.041, respectively), but not OS.

## DISCUSSION

MiRNAs show promise as leading molecular biomarkers to predict response to chemoradiation in locally-advanced rectal cancer. This is one of the only studies to correlate miRNA molecular profiles with both pCR and long-term clinical outcomes such as distant metastasis-free survival, relapse-free survival, and overall survival, as most previous studies in rectal cancer patients who received chemoradiation focused on simply endoscopic (primary tumor) or pathologic response. One recent study (the only other one to our knowledge), reported that miR-31 predicted pCR to chemoradiation and overall survival [25]. Unfortunately, we could not confirm previously identified miRNAs or identify any novel miRNAs to be significantly associated with pCR after false discovery rate correction. However, we did identify pre-therapy let-7b levels as significantly correlated with long-term outcomes, confirming the importance of let-7 family members as important for overall outcomes. In addition, we are only the second study to report changes in miRNA expression profiles before and after chemoradiation for rectal cancer. We determined miR expression patterns were significantly changed in their pre-chemoradiation state versus post-chemoradiation in residual tumor tissue in patients who were found to have incomplete response.

One of the miRs that was most up-regulated after therapy was miR-125b. This miR has been extensively studied in colorectal cancer [19, 26–28]. miR-125b appears to be up-regulated in colorectal cancer compared to normal tissue, and in addition, correlates with a poor prognosis, suggesting a pro-tumorigenic role [29]. Other studies have found elevated miR-125b expression levels to correlate with a poor response to chemoradiation [19, 26]. *In vitro* studies have identified possible targets of miR-125b to be p53, and BAK1 [27]. By inactivating these important proteins identified as important in the response to DNA damage, this data further supports miR-125b as having an oncogenic role and aids in the progression and therapeutic resistance of colorectal cancer. Our data found miR-125b levels to be six times higher after therapy in tumor tissue at the time of surgical resection. Due to its silencing of p53 and BAK1 (Bcl2L7), it may induce radioresistance by subverting the response that would normally promote apoptosis after chemoradiation-induced DNA damage.

We also identified that let-7b was up-regulated in post-therapy residual tumor tissue, suggestive of let-7b as another miRNA mediating survival. In our study, we found multiple let-7 family members including let-7b, let-7c, and let-7e, that were significantly up-regulated in post-treatment residual tumor tissue (1.4-fold, 2.6-fold, and 1.5-fold respectively). In addition, increased pre-therapy let-7b levels correlated with decreased DMFS, RFS, and a lower overall survival, suggesting that increased let-7b levels before therapy might be tied to a poor response to chemoradiation, and worse overall outcomes. Interestingly, the let-7 family have been linked to Ras signaling [30], with let-7 members negatively regulating Ras, which is somewhat counter-intuitive with our results. However, other studies have shown that higher expression of let-7b or let-7a can be associated with poor clinical outcomes [31–33], and inhibition of let-7b can reduce angiogenesis and tumor cell mobility in preclinical studies, providing evidence that let-7b has pro-tumorigenic activity.

Another set of miRs that were elevated in radioresistant tumors after therapy was the miR-99 family. Both miR-99a and miR-99b were significantly elevated after chemoradiation. Unlike miR-125b, elevated miR-99 levels in the pre-therapy tissue have been found to correlate with improved response to therapy. *In vitro* studies have identified Bcl-2 and mTOR as possible targets for miR-99, supporting that miR-99 inhibits anti-apoptotic/pro-survival pathways. In addition, one study found that miR-99a targets SNF2H/SMARCA5 and reduced BRCA1 localization to sites of DNA damage [34]. As a result, miR-99 family miRNAs may be able to reduce the rate and efficiency of DNA repair through both non-homologous end-joining and homologous recombination. Thus, miR-99 in the pre-therapy setting may be exerting tumor-suppressive and radiosensitizing effects. Our findings that miR-99 is elevated in post-chemoradiation residual tumor tissue, is somewhat counter-intuitive. Given its established role as pro-apoptotic, we would have hypothesized that this miRNA would have been expressed in lower levels in post-treatment surviving cells or residual tumor tissue. However, one study demonstrated that levels of miR-99 can be increased following radiation, which supports our findings [34]. In addition, some of the miRs that were most significantly downregulated with an FDR <0.0005 including miR-200, miR-203, and miR-196 have been previously been shown to be tumor suppressive [35–37]. Thus, these changes in miR expression may have contributed to cancer recurrence and failure of chemoradiation therapy.

The IPA of the differentially expressed miRNAs identified multiple pathways that may contribute to chemoradiation resistance, and may explain the survival of residual cancer cells. One pathway involved inhibition of the RAS family of GTPases, proteins that have been shown to correlate with radiation resistance in many types of cancers [38–43]. Another group of proteins identified in this network were Smad2 and Smad3, downstream proteins in the TGF-B pathway. Inhibition of the Smad proteins and therefore the TGF-B signaling pathway has been associated with a worse prognosis [44]. Another study showed that in colon cancer, proper Smad protein functionality is required in order for TGF-B to produce its apoptotic and growth inhibitory effects [45]. Alternate studies have shown that TGF-B signaling can promote tumor growth and survival through increased miR-181b [46]. In our study, we found miR-181b significantly increased after therapy (1.3-fold). Finally, Akt was another important protein identified in the IPA network. Akt is downstream of Ras signaling, and Akt signaling has also been shown to mediate resistance to radiation and chemoradiation via numerous mechanisms, by promoting accelerated DNA repair (e.g. non-homologous end-joining), preventing apoptosis by inhibition of Bad (a pro-apoptotic Bcl-2 family member), and also allowing for un-regulated progression through the cell cycle [47]. There is also evidence that up-regulated p38 MAPK activity decreases inflammation and the immune response to cancer, and therefore decreased MAPK p38 activity might correlate with more advanced cancer and metastasis [48]. In our analysis of residual tumor tissue, we found miR-146 levels were decreased after chemoradiation, and the IPA network analysis linked down-regulated miR-146 to the p38 MAPK pathway, suggesting that down-regulated miR-146 levels could increase p38 MAPK activity and subsequently increase proliferation and survival.

The second network contained several targets that are important in oncogenesis. ERBB2, the gene that codes for HER2/neu was identified along with significantly decreased miR-548 in radioresistant tissue. A main target for miR-548 is ERBB2. Therefore in our treatment-refractory tissue (pIR), our findings suggest that the proto-oncogenic HER2/neu pathway may be dis-inhibited due to loss of miR-548. In breast cancer, HER2/neu signaling promotes radioresistance- therefore its elevation in residual tumor tissue may be likewise contributing to radiation resistance in rectal cancer [49]. In addition, the 2^nd^ network demonstrated that miR-191 was found to be significantly decreased in residual tumor tissue. An important target of miR-191 is IL-1, which in turn induces IL-6 expression. The lower levels of miR-191 thus could potentially raise IL-1 and IL-6 levels, and a previous study has shown increased IL-6 levels post radiation to correlate with a worse prognosis [50]. In addition, the anti-apoptotic protein and pro-proliferative E2F1 transcription factor, as well as the tumor suppressor gene CDK2NA were linked to our miR expression profile in the second network analysis.

A third network was identified using IPA, this network only examined the miRs with an FDR <10^−4^, and thus represented a more stringent (or restrictive) miRNA analysis. TP53 was a major part of this network, as well as ZEB2 and Bcl6. miR-127 has been identified as a potent inhibitor of the anti-apoptotic protein Bcl6, and therefore may have tumor suppressive properties [51]. However, in our network, miR-127 was found to be up-regulated in the post treatment tissue, which is counter-intuitive with what we would have expected based on these published findings. miR-125b was up-regulated in post-therapy tissue, and the IPA showed a relationship between miR-125b and TP53. One study found miR-125b to be a potent inhibitor of TP53, and thereby inhibit apoptosis and increase cell proliferation [52]. TP53, which encodes p53, is crucial for the cellular response to ionizing radiation, and p53 functional loss through allelic loss, loss of heterozygosity, and p53 mutations are linked to radioresistance. The increased levels of miR-125b in our post-therapy tissues are consistent with miR-125b as a having a pro-oncogenic role and promoting survival through inhibition of p53 activity. ZEB2 is a protein that has been identified as important in the metastasis of colorectal cancer [53]. The same study found miR-141 and miR-200 could inhibit ZEB2 (a mediator of tumor cell invasiveness/metastasis). Our data found levels of these two miRs to be reduced in the post treatment tumor tissue, and support that ZEB2 would be significantly over-expressed in these cancer cells, which could contribute to tumor metastasis and progression.

The IPA downstream effects predictor analysis, which searches the literature for miRs associated with a specific response, identified several miRs associated with the event “proliferation of carcinoma cell lines.” These include some of the miRs previously discussed such as let-7, miR-145, miR-143, and miR-99a. Each of these has been associated with a decreased proliferation of carcinoma cell lines, and the fact that each was increased following treatment suggested that the treatment decreased cell proliferation. This is somewhat counterintuitive, since a number of studies in head and neck and cervical cancer (squamous cell carcinoma) have been shown clinically to develop the phenomenon called “accelerated re-population” [54–57]. However, a slower cell proliferation phenotype may be supported if there is a strong anti-tumor immune effect and/or inflammatory response which attenuates tumor growth. In addition, post-therapy scarring and anti-angiogenic effects from chemoradiation could further limit tumor cell proliferation and expansion.

Finally, we found increased let-7b levels were associated with worse clinical outcomes. To our knowledge, this study is among the first to correlate expression of individual miRs with clinical outcomes, instead of merely clinical response to therapy. Increased pre-therapy let-7b levels portended a lower overall survival, increased distant metastasis, and decreased relapse-free survival time. Other studies have found disparate results with regards to let-7b levels and clinical prognosis. A recent study of miRs that may predict clinical outcomes in colorectal cancer found no significant relationship between let-7b and clinical outcomes [31]. Increased let-7b levels were found to correlate with a worse prognosis in serous ovarian carcinoma [33], and to increase angiogenesis and tumor mobility in prostate cancer [32]. However, studies in renal cell carcinoma found *decreased* levels of let-7b to correlate with resistance to therapy [58]. The best studied target of let-7b is CDC34, a gene coding for the ubiquitin conjugating enzyme Cdc34, of the Skp1/cullin/F-box (SCF) complex [59]. The SCF complex is important in cell cycle regulation, as it inhibits Wee1, and therefore encourages progression from G2 into M phase. Therefore, high levels of let-7b stabilize Wee1 and prevent entry into M phase. Since Wee1 kinase inhibition is associated with attenuated G2/M cell cycle checkpoint activation after radiation and subsequent radiosensitization [60], it is possible that high levels of let-7b stabilize Wee1 and activate G2/M cell cycle arrest. This cell cycle arrest might thereby promote heightened DNA repair after chemoradiation, decreased post-mitotic death, and subsequent resistance to therapy.

In summary, a number of miRs were found to be significantly differentially expressed after chemoradiation in residual tumor tissue. Further investigation is warranted to determine if each of these differentially expressed miRNAs directly contributed to a lack of complete response, resistance to therapy, or if they were simply altered as a direct effect of radiation-induced tissue damage and thus possess more of a “bystander or passenger” role. To our knowledge, this is one of the first studies to correlate miRNAs with long-term clinical outcomes such as distant metastasis free survival, relapse-free survival and overall survival for patients with locally-advanced rectal cancer undergoing chemoradiation followed by surgery. In so doing, we identified let-7b as a poor prognostic miRNA in rectal cancer for patients undergoing chemoradiation. Additional clinical studies are warranted to validate our findings with let-7b and the differentially expressed miRNAs after chemoradiation. Finally, it will be critical to determine whether the individual miRs identified as persisting after chemoradiation are directly contributing to chemoradiation resistance using preclinical models.

## MATERIALS AND METHODS

### Patient selection

We developed a database of >100 consecutive patients with locally advanced rectal cancer (LARC; clinically T3-T4 and/or node-positive [N+]) treated with neo-adjuvant chemoradiation followed by surgical resection (typically 6-10 weeks after chemoradiation) at the Ohio State University Wexner Medical Center, from 2004 – 2011. We collected data pertaining to demographics, staging, tumor markers, treatment, pathology, and outcomes for each patient. Archival formalin-fixed paraffin embedded (FFPE) tumor tissue was identified from both diagnostic biopsy and surgical resection specimens for this study. We identified 40 patients with sufficient tissue for this analysis. This retrospective review was approved by the institutional review board.

### Isolation of total RNA

Hematoxylin & eosin stained slides of corresponding FFPE blocks were used to mark areas of viable, non-necrotic tumor for coring. Then, 1.75 mm cores were obtained from the FFPE blocks using disposable 14 gauge needles. RNA isolation was performed using the Norgen FFPE RNA Isolation kit. Briefly, paraffin cores were cut and trimmed of any excess paraffin using sterile scalpels, ground with Eppendorf-sized mortar pestels, and deparaffinized by incubating with xylene at 50°C for 10 minutes followed by two 100% ethanol washes and air-dried to remove all traces of ethanol. Lysates were prepared by adding digestion buffer and proteinase K and incubating at 55°C for 15minutes followed by 80°C for 15 minutes. Buffer RL and ethanol were added and the lysate was transferred to a column and centrifuged until entire lysate had passed through the column to bind the RNA to it. On-column DNA removal was performed to remove traces of genomic DNA contamination by DNAse I. The column was then washed thrice, spun dry and the RNA was eluted in 15 microliters of elution solution. Concentration was measured by Nanodrop 2000.

### NanoString miRNA profiling

The NanoString nCounter Human v3 miRNA Expression Assay was used to perform the microRNA profiling analysis. The assay measures 800 different microRNAs at the same time for each sample. 100 ng of total RNA were annealed with multiplexed DNA tags (miR-tag) and target specific bridges. Mature microRNAs were bound to specific miR-tags using a ligase enzyme and all the tags in excess were removed by an enzyme clean-up step. The tagged microRNAs product was diluted 1 to 5, and 5 ul was combined with 20 ul of the Reported Probes in hybridization buffer and 5 ul of Capture probes. The overnight hybridization (16 to 20 hours) at 65°C allowed the probes to complex in a sequence specific fashion with the targets. Probe excess was removed using two-step magnetic beads based purification on an automated fluidic handling system (nCounter Prep Station), and target/probe complexes were immobilized on the cartridge for data collection. The nCounter Digital Analyzer collected the data by taking images of immobilized fluorescent reporters in the sample cartridge with a CCD camera through a microscope objective lens. For each cartridge, a high-density scan encompassing 600 fields of view was performed.

### Statistical analysis

After filtering of the NanoString data from 800 miRNAs, 168 miRNAs remained for analysis. A miRNA is filtered out if more than 90% of samples have a log count less than the negative background where the background is the mean of the log2 negative background counts plus 1.5 times the standard deviation of the log2 negative background counts, and a sample is removed if more than 60% of miRNA probes fall below the background cutoff. The final filtered data is geometric mean normalized and log2 transformed. A binomial generalized linear model (GLM) was used to identify miRs associated with pCR. A linear mixed effect model specifying the paired samples as random effects was used to identify miRs differentially expressed before and after treatment. Clinical outcomes (DM: distant metastasis, LRC: local regional control, RFS: relapse free survival, OS: overall survival) were evaluated using a Cox proportional hazard test. Gender was not included in the model as it had no bearing on the clinical outcomes. miR expression was dichotomized about the geometric mean for generating survival curves and the Kaplan-Meier estimate. Multivariable analysis was performed by including T stage or N stage as a covariate in the cox proportional hazard test. pN and pT stage were each individually tested in each of the 4 clinical outcomes.

## SUPPLEMENTARY MATERIALS TABLES





## Abbreviations

CRT: chemoradiation
miR: microRNA
pCR: pathologic complete response
pIR: pathologic incomplete response
cCR: clinical complete response
NOM: non-operative management
LARC: Locally advanced rectal adenocarcinoma
MDS: multi-dimensional scaling
IPA: ingenuity pathway analysis
OS: overall survival
RFS: relapse-free survival
DMFS: distant metastasis free survival
pT: pathologic tumor stage
pN: pathologic nodal stage
LRC: local regional control
FFPE: formalin-fixed paraffin embedded
GLM: generalized linear model
