# Supplementary material for: MicroRNA molecular profiling identifies potential signaling pathways conferring resistance to chemoradiation in locally-advanced rectal adenocarcinoma
Source: Oncotarget. 2018 Jun 22;9(48):28951–64. doi: 10.18632/oncotarget.25652 (PMC6034754; doi:10.18632/oncotarget.25652)
Supplement: Supplementary file 1 [file oncotarget-09-28951-s001.pdf]

MicroRNA molecular profiling identifies potential signaling pathways conferring resistance to chemoradiation in locally-advanced rectal adenocarcinoma

SUPPLEMENTARY MATERIALS

Supplementary Table 1: miRNAs significantly (FDR <.05) up or downregulated following chemoradiation

See Supplementary File 1

Supplementary Table 2: miRs and published effect on carcinoma proliferation

miR	Fold change post treatment	Reported effect on carcinoma proliferation based on literature	Final functional prediction on carcinoma proliferation
let-7c	2.592	Decreases	Decreased
miR-145	4.133	Decreases	Decreased
miR-143	2.281	Decreases	Decreased
miR-19b	-2.352	Increases	Decreased
miR-99a	6.699	Decreases	Decreased
miR-93	-2.011	Increases	Decreased
miR-203a	-2.876	Decreases	Increased
miR-200c	-3.315	Unknown	Unknown

Column 2 shows the fold change after therapy in our dataset while column 3 shows the reported effect that increased expression of a miR would have on carcinoma proliferation from a published literature review. For example, let-7c was found to be increased after treatment, and the literature reports that increased let-7c levels correlate with decreased proliferation. Conversely, miR-19b was found to be *decreased* post treatment in our data, and increased miR-19b levels correlate with increased carcinoma proliferation, therefore with decreased miR-19b levels we would expect decreased proliferation. In conclusion, the changes observed in 6 of the 8 miRs (let-7c, 145, 143, 19b, 99a, 93) support that there is decreased carcinoma proliferation in the post-treatment samples.
