# Supplementary material for: MicroRNA molecular profiling identifies potential signaling pathways conferring resistance to chemoradiation in locally-advanced rectal adenocarcinoma
Source: Oncotarget. 2018 Jun 22;9(48):28951–64. doi: 10.18632/oncotarget.25652 (PMC6034754; doi:10.18632/oncotarget.25652)
Supplement: Supplementary file 2 [file oncotarget-09-28951-s002.docx]

| miRNA | Fold Change | FDR |
| --- | --- | --- |
| hsa-miR-34a-5p | 2.199188656 | 1.84E-07 |
| hsa-miR-4286 | 12.98313259 | 1.84E-07 |
| hsa-miR-125b-5p | 6.260112822 | 5.91E-07 |
| hsa-miR-99a-5p | 6.699449823 | 5.91E-07 |
| hsa-miR-127-3p | 2.550633533 | 1.49E-06 |
| hsa-miR-100-5p | 3.947519857 | 1.91E-06 |
| hsa-miR-200a-3p | -2.253806916 | 2.38E-06 |
| hsa-miR-193a-5p/miR-193b-5p | 2.287800621 | 3.65E-06 |
| hsa-miR-106b-5p | -1.759748523 | 1.43E-05 |
| hsa-miR-99b-5p | 1.73210474 | 1.43E-05 |
| hsa-miR-194-5p | -3.36788945 | 1.47E-05 |
| hsa-miR-192-5p | -2.313123086 | 1.66E-05 |
| hsa-miR-203a-3p | -2.875754271 | 1.66E-05 |
| hsa-miR-429 | -2.155943399 | 3.39E-05 |
| hsa-miR-196a-5p | -1.933841333 | 3.83E-05 |
| hsa-miR-141-3p | -2.017675602 | 6.06E-05 |
| hsa-miR-365a-3p/miR-365b-3p | 1.94670275 | 6.16E-05 |
| hsa-miR-497-5p | 1.366235129 | 6.85E-05 |
| hsa-miR-20a-5p/miR-20b-5p | -2.547660942 | 0.000104477 |
| hsa-miR-200b-3p | -3.094169783 | 0.000109126 |
| hsa-miR-19b-3p | -2.352366925 | 0.000136756 |
| hsa-miR-145-5p | 4.133015438 | 0.000136756 |
| hsa-miR-7-5p | -1.897706576 | 0.000137587 |
| hsa-miR-106a-5p/miR-17-5p | -2.564120757 | 0.000148813 |
| hsa-miR-199a-5p | 2.091316973 | 0.000152677 |
| hsa-let-7c-5p | 2.592246535 | 0.000275023 |
| hsa-miR-19a-3p | -2.025036858 | 0.000277047 |
| hsa-miR-382-5p | 2.068178519 | 0.000277047 |
| hsa-miR-215-5p | -1.911193853 | 0.000317434 |
| hsa-miR-30a-5p | 1.437256662 | 0.000317434 |
| hsa-miR-143-3p | 2.28094088 | 0.000382549 |
| hsa-miR-200c-3p | -3.314871889 | 0.000382549 |
| hsa-miR-135b-5p | -2.452655438 | 0.000402982 |
| hsa-miR-93-5p | -2.011291052 | 0.000449371 |
| hsa-miR-107 | -1.265727593 | 0.000750415 |
| hsa-miR-376c-3p | 1.695149102 | 0.001082979 |
| hsa-miR-409-3p | 1.736828887 | 0.001082979 |
| hsa-miR-125a-5p | 1.634875865 | 0.001630789 |
| hsa-miR-22-3p | 1.660188548 | 0.00171059 |
| hsa-miR-374b-5p | -1.421551401 | 0.001849667 |
| hsa-miR-146a-5p | -1.688791579 | 0.001849667 |
| hsa-miR-1915-3p | 1.97769481 | 0.001951611 |
| hsa-miR-95-3p | -1.348865608 | 0.002034119 |
| hsa-miR-1973 | -1.346323032 | 0.00400302 |
| hsa-miR-342-3p | 1.689484155 | 0.005000249 |
| hsa-miR-320e | 1.676842193 | 0.007846854 |
| hsa-miR-25-3p | -1.504905446 | 0.008539188 |
| hsa-miR-32-5p | -1.434264868 | 0.009137661 |
| hsa-miR-4488 | 1.774173203 | 0.010608372 |
| hsa-miR-1972 | -1.970152473 | 0.010608372 |
| hsa-miR-214-3p | 1.776155997 | 0.010608372 |
| hsa-miR-199b-5p | 1.868163736 | 0.014984207 |
| hsa-miR-28-3p | 1.486458766 | 0.016023921 |
| hsa-miR-191-5p | -1.457076346 | 0.016023921 |
| hsa-miR-15b-5p | -1.891351794 | 0.017752763 |
| hsa-miR-1268a | 1.806600907 | 0.018657015 |
| hsa-let-7e-5p | 1.531235535 | 0.019370963 |
| hsa-miR-1285-5p | -1.589066088 | 0.019370963 |
| hsa-let-7b-5p | 1.413233237 | 0.019839379 |
| hsa-miR-15a-5p | -1.555584396 | 0.019839379 |
| hsa-miR-181a-5p | 1.314006765 | 0.020221068 |
| hsa-miR-1-3p | 1.573519352 | 0.02449436 |
| hsa-miR-4455 | -1.419361886 | 0.029313496 |
| hsa-miR-92a-3p | -1.29165765 | 0.034589683 |
| hsa-miR-548y | -1.332045032 | 0.035612116 |
| hsa-miR-1246 | -1.782049916 | 0.035612116 |
| hsa-miR-130a-3p | 1.562010105 | 0.036942677 |
| hsa-miR-374a-5p | -1.628435343 | 0.049696369 |

**Supplementary Table 1.** **miRNAs significantly (FDR <.05) up or downregulated following chemoradiation.** 68 Mir’s were significantly up or downregulated following chemoradiation. A negative fold change indicates a decrease after treatment and a positive fold change indicates an increase following treatment.
